# Perfectionism and stereotype in plastic surgery

**DOI:** 10.1192/j.eurpsy.2022.842

**Published:** 2022-09-01

**Authors:** L. Hogea, L. Corsaro, T. Anghel

**Affiliations:** 1 “Victor Babes” University of Medicine and Pharmacy, Department Of Neurosciences, Timisoara, Romania; 2 Campus Bio Medico, Plastic Surgery, Roma, Italy

**Keywords:** attitude, beauty, stereotyping, cosmetic surgery, perfectionism

## Abstract

**Introduction:**

The concept of beauty has transformed through time and across the globe during specific events in history and continues to evolve.

**Objectives:**

This study will focus on how tendency toward perfectionism and stereotypes promoted by media influence beauty perception and the need of plastic surgery.

**Methods:**

In this study we examined factors influencing attitudes toward plastic surgery among 23 women with an average 35 years old and the data were collected through three questionnaire: The abbreviated multidimensional perfectionism scale (MPS) is a 30-item measure separated into two 15-item subscales: self-oriented perfectionism and socially prescribed perfectionism; The abbreviated perfectionistic self-presentation scale (PSPS) is a 20-item measure divided into two ten-item subscales: perfectionistic self-promotion and non-display of imperfection. Participants’ perceptions of media messages about appearance issues have been assessed using 30 items of the Sociocultural Attitudes toward Appearance Questionnaire-3 (SATAQ-3). Sociocultural attitudes toward appearance, physical appearance perfectionism were considered as predictors of tendency toward plastic surgery.

**Results:**

The results showed that there is significant positive association between perfectionism, the influence of mass media and increased women’ s likelihood of undergoing plastic surgery.

**Conclusions:**

Our findings suggest firstly that a greater perfectionist tendency and psychological investment in physical appearance predict more favorable attitudes toward plastic surgery. Perfectionists women may choose plastic surgery as part of their need of bodily perfection. Secondly, the choice of plastic surgery depended on sociocultural attitudes toward physical appearance.

**Disclosure:**

No significant relationships.

